# Macrolide resistance determinants among *Streptococcus pneumoniae* isolates from carriers in Central Greece

**DOI:** 10.1186/1471-2334-12-255

**Published:** 2012-10-11

**Authors:** Ioanna N Grivea, Alexia Sourla, Eleni Ntokou, Denise C Chryssanthopoulou, Alexandra G Tsantouli, George A Syrogiannopoulos

**Affiliations:** 1 Department of Pediatrics, University of Thessaly, School of Medicine, General University Hospital of Larissa, Biopolis, 411 10, Larissa, Greece

## Abstract

**Background:**

We sought to characterize the temporal trends in nasopharyngeal carriage of macrolide-resistant pneumococci during a period with increased heptavalent pneumococcal conjugate vaccine (PCV7) coverage in Central Greece.

**Methods:**

*Streptococcus pneumoniae* isolates were recovered from 2649 nasopharyngeal samples obtained from day-care center attendees in Central Greece during 2005–2009. A phenotypic and genotypic analysis of the isolates was performed, including the identification of macrolide resistance genes *mef*(A), subclasses *mef*(A) and *mef*(E), as well as *erm*(B).

**Results:**

Of the 1105 typeable *S*. *pneumoniae* isolates, 265 (24%) were macrolide-resistant; 22% in 2005, 33.3% in 2006, 23.7% in 2007, and 20.5% in 2009 (*P*=0.398). Among these macrolide-resistant pneumococci, 28.5% possessed *erm*(B), 24.3% *erm*(B)+*mef*(E), 41.8% *mef*(E), and 5.3% *mef*(A). A *mef* gene as the sole resistance determinant was carried by 31% of macrolide-resistant isolates belonging to PCV7 serotypes and 75.8% of the non-PCV7 serotypes. Across the 4 annual surveillances, pneumococci carrying *mef*(A) gradually disappeared, whereas serotype 19F isolates carrying both *erm*(B) and *mef*(E) persisted without significant yearly fluctuations. Among isolates belonging to non-PCV7 serotypes, macrolide-resistance was observed in those of serotypes 6A, 19A, 10A, 15A, 15B/C, 35F, 35A, and 24F. In 2009, ie 5 years after the introduction of PCV7 in our country, 59% of macrolide-resistant pneumococci belonged to non-PCV7 serotypes.

**Conclusions:**

Across the study period, the annual frequency of macrolide-resistant isolates did not change significantly, but in 2009 a marked shift to non-PCV7 serotypes occurred. Overall, more than half of the macrolide-resistant isolates possessed *erm*(B) either alone or in combination with *mef*(E). *erm*(B) dominated among isolates belonging to PCV7 serotypes, but not among those of non-PCV7 serotypes.

## Background

*Streptococcus pneumoniae* is a gram-positive bacterium commonly found in the human nasopharynx, in particular during early childhood [1,2]. In most individuals colonization is asymptomatic and does not evolve into disease [3,4]. In addition to the carriage state, *S*. *pneumoniae* is also a common human pathogen responsible for bacteremia, sepsis, and meningitis, as well as noninvasive disease, such as non-bacteremic pneumonia, otitis media, and sinusitis [4,5].

Resistance to erythromycin and other macrolides is common in *S*. *pneumoniae* isolates recovered from carriers and patients [6-14]. The major mechanisms of macrolide resistance in *S*. *pneumoniae* are target modification and drug efflux [15]. The genetic determinant conferring macrolide resistance by target modification is mainly *erm*(B) [16]. The *erm*(B) gene methylates the peptidyl transferase center of 23S rRNA, thereby conferring high-level resistance to 14-, 15- and 16-membered ring macrolides, lincosamides and streptogramin B (MLS_B_ phenotype) [15].

The second macrolide resistance mechanism is an efflux pump system encoded by *mef* and an ATP-binding cassette protein encoded by the *mel* gene [17]. The efflux mechanism confers resistance to 14- and 15-member macrolides only (M phenotype) [18,19]. Two main variants of *mef*, *mef*(E) and *mef*(A), which are approximately 90% identical at the nucleotide level and have been assigned to the same class of macrolide resistance determinants, *mef*(A) [20], are found in *S*. *pneumoniae *[21,22]. These two genes are located on different genetic elements [22,23]. *mef*(A) is located on the defective transposon Tn*1207*.*1 *[24], or the closely related Tn*1207*.*3 *[25], whereas *mef*(E) on “mega” element [17,26,27].

Greece has a high prevalence of antibiotic-resistant *S*. *pneumoniae *[28-30]. In our country, PCV7 became available in October 2004; it was officially introduced to children younger than 5 years of age in January 2006 and was reimbursed by 80% by the national health insurance system in June 2006. The aim of the present investigation was to study trends in carriage of macrolide-resistant pneumococci and to analyze their antibiotic susceptibility, serotypes, and macrolide resistance determinants. The isolates were collected in a prospective study conducted among day-care center attendees in Central Greece between 2005 and 2009.

## Methods

### Study population

Nasopharyngeal specimens were obtained from children attending day-care centers in Larissa, Volos, Trikala, and Karditsa, the 4 largest cities of Central Greece during an approximately three-month period in 2005, 2006, 2007, and 2009. In the annual surveillance, 1 sample was obtained from each child. Information regarding the participant’s PCV7 vaccination status was collected. The research protocol was approved by the Ethics Committee of the General University Hospital of Larissa. Informed consent was obtained from one of the parents of each attendee.

### Vaccine schedule

The Hellenic National Committee for Immunization Programs recommends PCV7 for routine administration as a 4-dose series for infants at 2, 4, 6, and 12 to 18 months of age. Catch-up immunization is recommended for all children up to 59 months of age [31].

### Laboratory procedures

Specimens of nasopharyngeal secretions were obtained pernasally using sterile swabs on flexible shafts with calcium alginate fiber tips (Fisher Scientific, Pittsburgh, Philadelphia, USA). Swabs were placed in Amies transport medium (TGV, Sanofi Diagnostic Pasteur, Marne la Coquette, France) after sampling and were transferred to the Laboratory of the Division of Pediatric Infectious Disease of the University of Thessaly, where isolation, identification and susceptibility testing of the *S*. *pneumoniae* isolates were performed as previously described [32]. The maximum delay between collection and cultivation was 7h.

The swabs were plated onto Columbia agar plates supplemented with 5% defibrinated horse blood, 10 μg of colistin sulfate and 15 μg of nalidixic acid per milliliter. The plates were incubated at 35°C in an atmosphere supplemented with 5% CO_2_ for 24–72 h. Phenotypic characteristics (morphology and α-hemolysis) were used for the presumptive identification of pneumococci. Pneumococcal identification was confirmed by optochin susceptibility and bile solubility assays. When suspected pneumococcal colonies with more than one morphology were observed, each type was purified for further testing.

Susceptibility testing to various antimicrobial agents representing different classes of antibiotics was performed on Mueller-Hinton agar supplemented with 5% defibrinated horse blood, as follows. *S*. *pneumoniae* isolates were tested for susceptibility to erythromycin and clindamycin by both the disk diffusion method and the E-test method (AB Biodisk, Solna, Sweden). Isolates were screened for penicillin resistance using 1 μg oxacillin disks. If the oxacillin inhibition zone was <20 mm, minimal inhibitory concentration (MIC) to penicillin was determined by the E-test method. Susceptibility to quinolones was determined by the E-test method. Isolates were tested with levofloxacin except for isolates recovered in 2005 that were tested with ciprofloxacin. Finally, susceptibility to chloramphenicol, tetracycline, and trimethoprim-sulfamethoxazole (TMP-SMZ) was determined by the disk diffusion method. For susceptibility testing, plates with the antibiotic disks and E-test strips were incubated in 5% CO_2_. The susceptibility breakpoints of the Clinical and Laboratory Standards Institute (CLSI) [33] and the European Committee on Antimicrobial Testing (EUCAST) [34] were used to classify organisms as susceptible, intermediate or resistant to the studied antibiotics. The oral penicillin V susceptibility breakpoints of CLSI were applied since in pediatric infections the treatment is mainly oral: ≤0.06 μg/ml, susceptible; 0.12–1 μg/ml, intermediate; and ≥2 μg/ml, resistant. The benzylpenicillin susceptibility breakpoints of EUCAST for infections other than meningitis were used: ≤0.06 μg/ml, susceptible; 0.12–2 μg/ml, intermediate; and >2 μg/ml, resistant. Pneumococci were defined as resistant to ciprofloxacin if their ciprofloxacin MICs were ≥4 μg/ml. An isolate was defined as multidrug resistant (MDR) when it was resistant to ≥3 antibiotic classes. Penicillins, cephalosporins, and carbapenems were considered a single class.

The macrolide resistance phenotypes were determined on the basis of the pattern of susceptibility to erythromycin and clindamycin and confirmed by the double disk diffusion test using erythromycin and clindamycin disks (BBL, Cockeysville, MD). Specifically, 15 μg erythromycin and 2 μg clindamycin disks were placed 16 mm apart. Induction was present when the zone of inhibition around the clindamycin disk was blunted on the side next to the erythromycin disk.

### Detection and analysis of the *erm*(B) and *mef* genes

Bacterial DNA was extracted by using the QIAamp DNA Mini kit (QIAGEN, Hilden, Germany). The presence of macrolide resistance genes was detected by PCR as described previously [35]. In summary, we amplified the genes by PCR and analyzed the amplified DNA products by agarose gel electrophoresis. For *erm*(B) we used the primer pair 5’-CGA GTG AAA AAG TAC TCA ACC-3’ and 5’-GGC GTG TTT CAT TGC TTG ATG-3’ [36] and for *mef* gene the primer pair 5’-GCGTTTAAGATAAGCTGGCA-3’ and 5’-CCTGCACCATTTGCTCCTAC-3’ [22]. In order to discriminate between *mef*(A), subclasses *mef*(A) and *mef*(E), PCR restriction fragment length polymorphism analysis was performed, as suggested by Oster et al. [21]. The 1743-bp PCR product was digested with the BamHI or the DraI restriction enzyme. In *mef*(A) there is one BamHI site, so restriction generates two fragments of 1,340 and 403 bp, while in *mef*(E) there are no BamHI restriction sites. Restriction of *mef*(A) with DraI yields two fragements of 1,493 and 250 bp, respectively, while restriction of *mef*(E) yields three fragments of 782, 711, and 250 bp.

### Capsule serotyping

Serotype determination of pneumococci, including serotype 6C, was performed at our Laboratory in Larissa by using Pneumotest-Latex and by the capsular swelling method using pneumococcal type/group and/or factor antisera from Statens Serum Institut (SSI, Copenhagen, Denmark). We followed the SSI guidelines for serotyping.

### Statistical analysis

An attendee was defined as age-appropriately vaccinated if at sampling the child had received all the PCV7 doses recommended for the age at initiation of immunization [37]. A dose of PCV7 vaccine was counted if it had been received at least 30 days before the sampling date. Pneumococcal isolates were classified as PCV7 serotypes, non-PCV7 serotypes and nontypeable.

To assess the 4 groups of attendees enrolled during the 2005, 2006, 2007, and 2009 surveillance, categorical parameters were compared using the χ^2^ for trend. For the assessment of 2 groups, categorical parameters were compared using 2-sided Fisher exact test. The statistical analysis was performed using SPSS version 13.0. An effect was considered significant when *P*<0.05.

## Results

### Population and samples

Between February 28, 2005 and May 19, 2009, cultures were obtained from 2649 children aged 13 to 76 months (median age: 48 months). There were no children younger than 13 months old attending the studied day-care centers. The characteristics of children at the time of sampling are presented in Table 1. Description of the day-care centers has been published previously [8,30]. Of the 2649 children, 1196 (45.1%) were identified as carriers of *S*. *pneumoniae*. Forty-six attendees carried two different pneumococcal isolates. Of the totally 1242 pneumococcal isolates, 1105 (89%) were typeable. The present analysis was based on this collection of typeable isolates.

**Table 1:** **Characteristics of children at the time of enrollment** (**N**=**2649**)

**Characteristic**	**Year of surveillance**				***P***
	**2005**	**2006**	**2007**	**2009**	
Time-period of enrollment	February 28 to June 7	February 2 to April 13	February 26 to May 17	February 24 to May 19	
PCV7 in the National Immunization Program	--	+	+	+	
PCV7 reimbursed	--	--	+	+	
No. of enrolled children	769	494	566	820	
Age, median (range), months	49 (15–76)	46.5 (13–70)	46 (13–73)	48 (14–72)	—
Male gender	417 (54.2)^a^	248 (50.2)	297 (52.5)	427 (52.1)	0.529
Antibiotic use in the preceding 3 months	433/764^b^ (56.7)	260/489 (53.2)	284/566 (50.2)	459/818 (56.1)	0.647
Vaccinated with ≥1 dose of PCV7	99 (12.9)	161 (32.6)	397 (70.1)	783 (95.5)	<**0**.**001**
Age-appropriately vaccinated	92 (12)	150 (30.4)	351 (62)	668 (81.5)	<**0**.**001**
*Streptococcus pneumoniae* carriage					
per age-group, months					
13 − 23	12/21 (57.1)	11/22 (50)	18/26 (69.2)	13/18 (72.2)	0.176
24 – 35	74/121 (61.2)	34/69 (49.3)	50/99 (50.5)	43/100 (43)	**0**.**01**
36 – 47	130/231 (56.3)	78/185 (42.2)	84/183 (45.9)	118/279 (42.3)	**0**.**006**
48 – 59	101/240 (42.1)	70/188 (37.2)	109/208 (52.4)	132/349 (37.8)	0.721
≥60	53/156 (34)	13/30 (43.3)	25/50 (50)	28/74 (37.8)	0.273
Overall	370 (48.1)	206 (41.7)	286 (50.5)	334 (40.7)	**0**.**036**

^a^Number in parentheses is percent, unless otherwise indicated.

^b^The denominator indicates the number of children with available information on recent antibiotic use.

### Carriage of macrolide-resistant pneumococci

**Figure 1:**
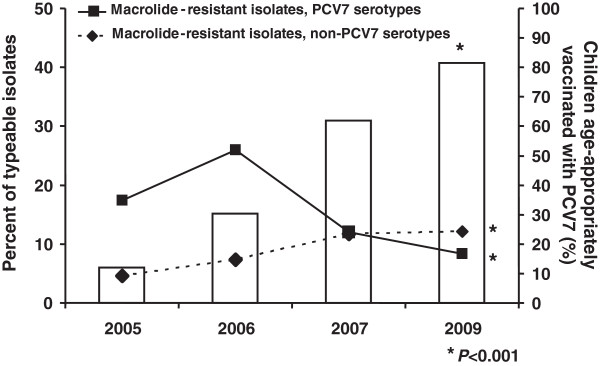
**Serotype distribution of macrolide**-**resistant nasopharyngeal pneumococcal isolates and percentage of attendees age**-**appropriately vaccinated with PCV7 at the time of surveillance.**

From 2005 to 2009, 265 (24%) of the 1105 typeable *S*. *pneumoniae* isolates were macrolide-resistant. Across the 4 surveillance periods, the proportion of macrolide-resistant isolates did not change significantly. Specifically, their frequencies were 22% (77 of 350) in 2005, 33.3% (64 of 192) in 2006, 23.7% (63 of 266) in 2007, and 20.5% (61 of 297) in 2009 (*P*=0.398). However, across the 4 sampling periods a significant decrease was noted in macrolide-resistant isolates belonging to PCV7 serotypes (from 17.4% to 26%, 12% and 8.4%; *P*<0.001) and a significant increase in the proportion of macrolide-resistant isolates belonging to non-PCV7 serotypes (from 4.6% to 7.3%, 11.7% and 12.1%; *P*<0.001) (Figure 1).

Among macrolide-resistant pneumococci, the proportion of isolates belonging to non-PCV7 serotypes was 20.8% (16 of 77) in 2005, 21.9% (14 of 64) in 2006, 49.2% (31 of 63) in 2007, and 59% (36 of 61) in 2009 (*P*<0.001).

During the first 2 annual surveillances, PCV7 serotypes 19F, 23F, 14, and 6B accounted for 60 (77.9%) of the 77 macrolide-resistant isolates in 2005 and 50 (78.1%) of the 64 in 2006. In contrast, during the 2009 surveillance, it was non-PCV7 serotypes 6A, 19A, 15A, and 15B/C that accounted for 33 (54.1%) of the 61 macrolide-resistant pneumococci.

### Macrolide resistance determinants, phenotypes, and co-resistance

Of the 265 macrolide-resistant pneumococci, 263 were studied for the presence of macrolide resistance determinants (Table 2). Seventy-five (28.5%) carried the *erm*(B) gene, 64 (24.3%) the *erm*(B)+*mef*(E) genes, 110 (41.8%) *mef*(E), and 14 (5.3%) *mef*(A) (Table 2). All mef(A)- or *mef*(E)-positive *S*. *pneumoniae* isolates exhibited the M-phenotype. All *erm*(B)- and dual *erm*(B)+*mef*(E)-positive isolates showed the constitutive MLS_B_ phenotype.

**Table 2:** Macrolide resistance determinant according to the serotype

**Serotype**	**No**. **of isolates**	**Macrolide resistance determinant**			
		***erm*(B)**	***erm*(B)+*mef*(E)**	***mef*(E)**	***mef*(A)**
6A	69	0	0	64 (92.8)	5 (7.2)
6B	12	11 (91.7)^a^	0	1 (8.3)	0
9V	1	0	0	1 (100)	0
10A	5	5 (100)	0	0	0
14	17	8 (47.1)	0	0	9 (52.9)
15A	3	3 (100)	0	0	0
15B	3	3 (100)	0	0	0
15C	1	1 (100)	0	0	0
19A	8	7 (87.5)	0	1 (12.5)	0
19F	108	3 (2.8)	64 (59.3)	41 (38)	0
23F	30	30 (100)	0	0	0
24F	1	1 (100)	0	0	0
35A	2	0	0	2 (100)	0
35F	3	3 (100)	0	0	0

^a^Number in parentheses, percent.

MICs to erythromycin of *mef*(A)-positive isolates ranged from 8 to 64 μg/ml (MIC_50_=32 μg/ml; MIC_90_=64 μg/ml), *mef*(E)-positive from 1 to 64 μg/ml (MIC_50_=4 μg/ml; MIC_90_=8 μg/ml), *erm*(B)-positive from 4 to 256 μg/ml (MIC_50_=256 μg/ml; MIC_90_=256 μg/ml), and dual *erm*(B)+*mef*(E)-positive was 256 μg/ml (MIC_90_=256 μg/ml).

**Figure 2:**
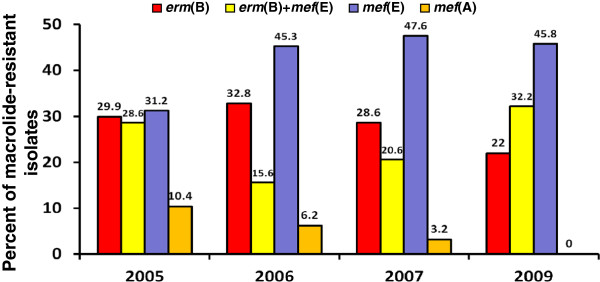
Frequencies of macrolide resistance determinants according to the year of surveillance.

**Figure 3:**
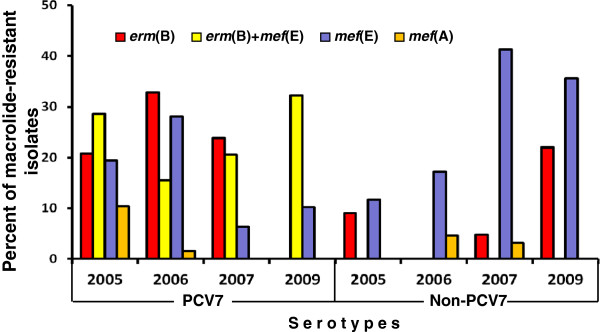
**Frequencies of macrolide resistance determinants among isolates belonging to PCV7 and non**-**PCV7 serotypes according to the year of surveillance.**

A significant decline in *mef*(A) isolates was noted across the study period (Figure 2). Specifically, *mef*(A)-positive pneumococci of PCV7 serotypes accounted for 10.4% of the macrolide-resistant isolates in 2005, 1.6% in 2006, and 0% in 2007 and 2009 (*P*<0.001), whereas *mef*(A)-positive isolates of non-PCV7 serotypes accounted for 0% of the macrolide-resistant isolates in 2005, 4.7% in 2006, 3.2% in 2007, and 0% in 2009 (*P*=0.993) (Figure 3).

Initially, the *mef*(E) gene was common among isolates of PCV7 serotypes, but since 2007 its frequency is higher among pneumococci of non-PCV7 ones (Figure 3). Specifically, *mef*(E)-positive isolates belonging to PCV7 serotypes accounted for 19.5% of the macrolide-resistant isolates in 2005, 28.1% in 2006, 6.3% in 2007, and 10.2% in 2009 (*P*=0.018), while *mef*(E)-positive pneumococci belonging to non-PCV7 serotypes accounted for 11.7% of the macrolide-resistant isolates in 2005, 17.2% in 2006, 41.3% in 2007, and 35.6% in 2009 (*P*<0.001).

The *erm*(B) gene was common initially, from 2005 through 2007, among isolates belonging to PCV7 serotypes and in 2009 among those of non-PCV7 serotypes (Figure 3). Specifically, *erm*(B)-positive isolates belonging to PCV7 serotypes accounted for 20.8% of the macrolide-resistant isolates in 2005, 32.8% in 2006, 23.8% in 2007, and 0% in 2009 (*P*=0.003), while *erm*(B)-positive isolates of non-PCV7 serotypes accounted for 9.1% of the macrolide-resistant pneumococci in 2005, 0% in 2006, 4.8% in 2007, and 22% in 2009 (*P*=0.013).

**Figure 4:**
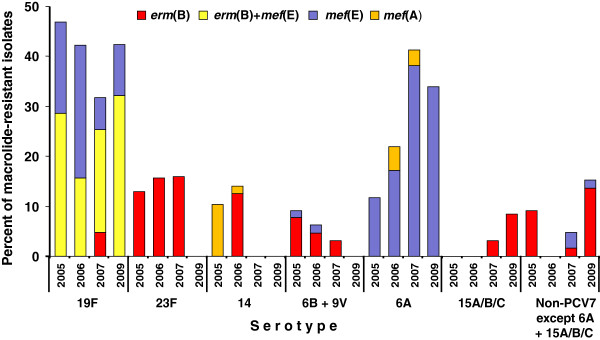
Serotype specific macrolide resistance determinants according to the year of surveillance.

The *erm*(B)+*mef*(E)-positive isolates belonged to serotype 19F (Table 2) and accounted for 28.6% of the macrolide-resistant isolates in 2005, 15.6% in 2006, 20.6% in 2007, and 32.2% in 2009 (*P*=0.638) (Figure 4).

Among the macrolide-resistant pneumococcal isolates recovered in 2005 and 2006, 53.9% carried the *erm*(B) gene alone or in combination with *mef*(E). In 2009, 54.2% of the macrolide-resistant isolates possessed *erm*(B) alone or in combination with *mef*(E) (Figure 2).

Co-resistance rates among the 263 isolates with molecular evaluation are presented in Table 3. According to both CLSI and EUCAST breakpoints, nonsusceptibility to one or more other antimicrobial agents was found in 248 (94.3%) of the 263 erythromycin-resistant isolates, constituting 16 different resistance patterns. Of these 248 isolates, 92.3% were nonsusceptible to penicillin, 70.2% (CLSI breakpoints) and 68.5% (EUCAST breakpoints) to TMP-SMZ, 63.7% to tetracycline, 56% to clindamycin and 16.9% to chloramphenicol.

**Table 3:** **Co**-**resistance among macrolide**-**resistant isolates of *Streptococcus pneumoniae***, **2005**-**2009**

**Antibiotic nonsusceptibility**	***erm*(B)**		***erm*(B)+*mef*(E)**		***mef*(E)**		***mef*(A)**
	**(n=75)**		**(n=64)**		**(n=110)**		**(n=14)**
	**CLSI**	**EUCAST**	**CLSI**	**EUCAST**	**CLSI**	**EUCAST**	**CLSI/EUCAST**
Penicillin-nonsusceptible							
MIC_50/90_ (MIC range)	1/2 (0.125-2)		2/4 (0.25-4)		0.25/2 (0.125-4)		
Intermediate	43 (57.3)^a,b^	58 (77.3)^c^	21 (32.8)	52 (81.2)	85 (77.3)	105 (95.5)	0
Resistant	15 (20)	0	43 (67.2)	12 (18.8)	22 (20)	2 (1.8)	0
Clindamycin	75 (100)	75 (100)	64 (100)	64 (100)	0	0	0
Tetracycline	55 (73.3)	55 (73.3)	63 (98.4)	63 (98.4)	40 (36.4)	40 (36.4)	0
Chloramphenicol	41 (54.7)	41 (54.7)	1 (1.6)	1 (1.6)	0	0	0
TMP-SMZ	62 (82.7)	61 (81.3)	64 (100)	64 (100)	48 (43.6)	45 (40.9)	0
MDR	74 (98.7)	73 (97.3)	64 (100)	64 (100)	48 (43.6)	46 (41.8)	0

^a^Number in parentheses is percent, unless otherwise indicated.

^b^Oral penicillin V breakpoints.

^c^Benzylpenicillin breakpoints (infections other than meningitis).

TMP-SMZ: trimethoprim-sulfamethoxazole; MDR: multidrug-resistant.

Multidrug-resistance was significantly more frequent among isolates carrying *erm*(B), either alone or in combination with *mef*(E), than among isolates harboring *mef*(E) or *mef*(A) alone (*P*<0.001) (Table 3). Pneumococci possessing *erm*(B) expressed a total of 11 different resistance patterns. Macrolide-resistant serotype 14 isolates harboring *erm*(B) and exhibiting penicillin nonsusceptibility (MICs 1–2 μg/ml; MIC_90_=2 μg/ml) were found in the 2006 surveillance in 3 day-care centers. Finally, isolates carrying either *mef*(E) or *mef*(A) expressed five different resistance patterns.

Fourteen macrolide-resistant isolates had an MIC to penicillin of 4 μg/ml; 12 (18.8%) of the 64 isolates with *erm*(B)+*mef*(E) and 2 (1.8%) of the 110 *mef*(E)-positive pneumococci (Table 3).

Of the 265 macrolide-resistant pneumococcal isolates, 188 were tested with levofloxacin and all of them were found to be susceptible (MICs 0.25–1 μg/ml; MIC_50_=0.5 μg/ml; MIC_90_=1 μg/ml). Among the remainder 77 isolates tested with ciprofloxacin, we did not identify any ciprofloxacin-resistant isolates. These 77 pneumococci had ciprofloxacin MICs from 0.25 to 1 μg/ml (MIC_50_=1 μg/ml; MIC_90_=1 μg/ml).

## Discussion

In Greece, an increase in the rate of macrolide resistance among *S*. *pneumoniae* occurred after the introduction of newer macrolides in the 1990s and their extensive use thereafter. We have published the phenotypical and molecular analysis of the macrolide-resistant pneumococci recovered from young carriers in different geographic locations of Greece between 1995 and 1999 [32,38,39]. The overall rate of macrolide-resistant *S*. *pneumoniae* nasopharyngeal isolates was 18%, while these isolates belonged mainly to serotypes 23F, 6B, 19F, and 14 (in order of decreasing frequency). Subsequently, studies on clinical as well as colonizing isolates from Greece [R] have reported significantly higher rates of macrolide resistance (up to ~50%) than that found in our initial studies [28,29,40]. The highest rate of macrolide resistance has been reported in pneumococci recovered from children with non-invasive infections, particularly acute otitis media [28].

Across the 4 surveillance periods of the present study and in parallel to an increase in the number of children who were immunized with PCV7, the frequency of the macrolide-resistant isolates did not change significantly. Overall, 24% of the typeable *S*. *pneumoniae* isolates were macrolide-resistant. This result is in line with a study on carriage among day-care attendees in Lisbon [41]. However, in 2009 a major shift in the serotype distribution of macrolide-resistant isolates occurred. Macrolide-resistant isolates of non-PCV7 serotypes replaced those belonging to PCV7 ones. This shift is in accordance with a recent French study [7]. Vaccination against 7 serotypes of *S*. *pneumoniae* has led to the near extinction of vaccine serotypes in both asymptomatic carriage and disease [7,11,41]. In carriage, vaccine serotypes have been replaced by nonvaccine serotypes. Clonal expansion and/or serotype switching contribute to this replacement [42].

Overall, more than half (52.9%) of our macrolide-resistant isolates possessed *erm*(B) either alone or in combination with *mef*(E). A significant association was found between PCV7 serotypes and the presence of *erm*(B), either alone or in combination with *mef*(E) on the one hand and non-PCV7 serotypes and a *mef* gene as the sole resistance determinant on the other. Among pneumoccoci harboring a *mef* gene as the sole resistance determinant, the ratio of *mef*(E)- to *mef*(A)-positive isolates was 7.9:1. This ratio was significantly reversed from the one that we observed among carriers during 1995–1999, which was 1:2.3 [39], and the 1:5 ratio reported from Germany during 2005–2006 [23].

As an overall concept, drug efflux mediated by *mef* genes has been the most common mechanism in strains of *S*. *pneumoniae* in North America, whereas in most of the European countries and the Far East, the prevalent mechanism has been rRNA methylation encoded by *erm*(B) [9]. Nevertheless, this pattern of macrolide resistance determinants is not static and may be changing due to clonal spread of *S*. *pneumoniae* of certain serotypes and horizontal transfer of *mef* elements among streptococci [23,43]. Actually, in recent papers, *mef*(A) was the predominant macrolide resistance determinant in Norway (2001–2005) [44] and Germany (2005–2006) [23], whereas increased frequency of *erm*(B) as well as of the dual combination was found in the United States (2005–2008) [13,45].

In the pre-PCV7 period, pneumococci of serotype 14 contributed significantly to macrolide resistance. Among children, serotype 14 had a higher frequency in invasive disease than observed in carriage and non-invasive disease [28]. We have previously reported the circulation of *mef*(A)-positive macrolide-resistant, penicillin-susceptible serotype 14 isolates with a genotype identical to the international clone England-9^14^ among young carriers in Greece [39]. Similar isolates continued to circulate in our area during the first 2 years of the present study. In addition, in 2006 we found macrolide-resistant serotype 14 isolates possessing *erm*(B) and exhibiting penicillin-nonsusceptibility. Following the immunization with PCV7, a rapid decrease in the circulation of serotype 14 was noted and it is no longer a major macrolide-resistant serotype in Greece as well as in other countries [46,47].

This study reports a high frequency of serotype 19F pneumococci with both *erm*(B) and *mef*(E) recovered from carriers in several day-care centers in Central Greece across all sampling periods. Since 2001, isolates with the dual resistance mechanism have been increasingly reported from many parts of the world [13,23,45,48-50]. They have mainly been isolated from carriers or patients with non-invasive disease, particularly acute otitis media [45,50]. Worldwide, most isolates with the dual resistance mechanism belong to serotypes 19F or 19A [45,48]. Although serotype 19F is represented in the PCV7 vaccine, it affords low levels of protection against upper respiratory infections such as acute otitis media [51] and has been shown to be the least immunogenic of the vaccine serotypes [52]. Moreover, little evidence shows that 19F provides cross-protection against serotype 19A. In Greece, antibiotic pressure may have also contributed to the persistence of these MDR isolates. In our country, isolates with the dual resistance mechanism in a low frequency were identified for the first time among carriers in Athens during 2003 [29]. In the present study, which differentiated between *mef*(A) subclasses *mef*(A) and *mef*(E), only isolates carrying the *mef*(E) gene, but not *mef*(A), with *erm*(B) were observed, underscoring the different genetic background of *mef*(E) and *mef*(A). Our findings are in line with a recent study from the USA [45]. *S*. *pneumoniae* isolates possessing the *mef*(A) subclass *mef*(A) gene, carried on transposon Tn*1207*.*1*, in combination with *erm*(B) have been described in a paper from Australia [53].

## Conclusions

From 2005 to 2009, the annual rate of macrolide-resistant colonizing isolates did not change significantly. Overall, more than half (52.9%) of the macrolide-resistant isolates harbored the *erm*(B) gene either alone or in combination with *mef*(E). Multidrug resistance was significantly more common among the *S*. *pneumoniae* isolates carrying *erm*(B) than among those possessing the *mef*(E) gene as the single macrolide resistance determinant. Across the 4 sampling periods, the circulation of isolates possessing the *mef*(A) gene gradually disappeared. Marked changes were observed in the most recent surveillance, as the PCV7 coverage increased. In 2009, a shift to macrolide-resistant pneumoccoci belonging to non-PCV7 serotypes was noted. Serotypes 6A, 19A, 15A, and 15B/C accounted for 54.1% of macrolide-resistant pneumoccoci. Further trends in the carriage of macrolide resistance determinants among pneumococci belonging to non-PCV7 serotypes in the post-PCV7 period remain to be addressed by future surveillance studies.

## Competing interests

The authors declare that they have no competing interests.

## Authors’ contributions

ING was responsible for the bacteriological and molecular analyses. AS and EN participated in the laboratory analyses. DCC and AGT participated in the data analysis. GAS conceived the study and performed the data analysis. ING and GAS drafted the manuscript. AS, EN, DCC and AGT helped to draft the manuscript. All the authors read and approved the final manuscript.

## Pre-publication history

The pre-publication history for this paper can be accessed here:

http://www.biomedcentral.com/1471-2334/12/255/prepub
